# Quality of life after gastric bypass surgery in patients with type 2 diabetes: patients’ experiences during 2 years of follow-up

**DOI:** 10.1186/s13098-020-00597-1

**Published:** 2020-10-12

**Authors:** Petros Katsogiannos, Eva Randell, Magnus Sundbom, Andreas Rosenblad, Jan W. Eriksson, Janeth Leksell

**Affiliations:** 1 Departments of Medical Science, Clinical Diabetes and Metabolism Sciences, Uppsala University, Uppsala, Sweden; 2 School of Education, Health and Social Studies, Dalarna University, Falun, Sweden; 3 Department of Surgical Sciences, Uppsala University, Uppsala, Sweden

**Keywords:** RYGB, Quality of life, Type 2 diabetes

## Abstract

**Background:**

To examine the effects of gastric bypass surgery on health-related quality of life (HRQoL) in obese patients with type 2 diabetes, and to investigate their experiences of life adjustments using quantitative and qualitative methods.

**Methods:**

Thirteen patients with type 2 diabetes and obesity, (body mass index, BMI > 30 kg/m^2^), participating in a randomized clinical trial, completed this sub-study. HRQoL was evaluated before, and at 6 months and 2 years after gastric bypass surgery, using the RAND- 36-item health survey. At 2 years, interviews for in-depth analysis of HRQoL changes were performed.

**Results:**

Significant improvement was observed from baseline to 6 months for 2 of the eight health concepts, general health, and emotional well-being. At 2 years, improvements were also seen in physical functioning, energy/fatigue, as well as sustained improvements in general health and emotional well-being. Multiple regression analyses showed mostly non-significant associations between the magnitude of decrease in weight, BMI, and HbA1c during follow-up and improvement in HRQoL. The analyses from qualitative interviews supported a common latent theme “Finding a balance between the experience of the new body weight and self-confidence”.

**Conclusions:**

The improved HRQoL after gastric bypass surgery in obese patients with type 2 diabetes was not explained specifically by the magnitude of weight loss, but rather by the participants achieving a state of union between body and consciousness.

*Trial registration* ClinicalTrials.gov Identifier NCT02729246. Date of registration 6 April 2016 – Retrospectively registered https://clinicaltrials.gov/ct2/show/NCT02729246?term=bariglykos&draw=2&rank=1

## Background

The global prevalence of obesity and type 2 diabetes mellitus (T2DM) is growing and is widely recognized as one of the most challenging threats to public health [1]. Despite improvements in pharmacotherapy, fewer than 50% of patients with established T2DM achieve and maintain adequate thresholds for glycaemic control [2], which in turn contributes to serious health consequences and health care costs. Furthermore, health-related quality of life (HRQoL) is also negatively affected in obese patients with T2DM as these patients suffer from specific problems relating to pain, mobility, and discomfort as well as from increased cardiovascular risk [3].

Many observational studies as well as clinical trials have shown that weight loss surgery rapidly improves glycaemic control and cardiovascular risk factors in obese patients with T2DM [4, 5]. Roux-en-Y gastric bypass (RYBG), one of the most used metabolic procedures worldwide, is known to alleviate obesity-related diseases and improve quality of life by inducing large, and sustainable, weight loss [6]. The exact working mechanism is unknown, but reduced peroral intake due to exclusion of the stomach and improved glucose handling, when nutrients are entered the gastrointestinal system below the pancreas, are of great importance.

Previous quantitative research has focused on the clinical outcomes of metabolic surgery, whereas qualitative research has provided detailed accounts of the psychosocial impacts of the surgery [7, 8]. A recently published meta-analysis showed mixed results of HRQoL when comparing metabolic surgery with medical treatment among obese subjects with T2DM. One shortcoming with the included studies is their heterogeneity when measuring HRQoL and treatment satisfaction [9]. In general, metabolic surgery results in large improvements in physical health, while there are mixed results concerning mental health [10].

As the number of obese people with diabetes who seek metabolic surgery increases, more research is needed into the experiences of patients who have undergone the surgery [11]. Patient-Reported Outcomes Measures (PROM), increasingly used to understand patient-focused outcomes from provided care [12], can be measured by various questionnaires. Several types of PROM, either generic or disease-specific, exist. A widely used generic HRQoL measure across populations, and in diabetes research, is the 36-item Short-Form Health Survey (SF-36), developed by the Medical Outcomes Study [13, 14]. An identical version of the questionnaire is currently available in Swedish; the RAND 36-item Health Survey [15].

In this mixed-methods study, the effects of RYGB on the HRQoL of obese patients with T2DM as well as their experiences with subsequent life adjustments were assessed. Besides, we conducted thorough qualitative interviews to understand how the participants expressed their HRQoL after surgery. This study is a part of a randomized clinical trial in which we focus on physiological changes after surgery [16, 17].

## Methods

Patients with T2D with a duration of no more than 10 years, treated with oral antidiabetic drugs or GLP-1 analog, were recruited. Exclusion criteria included treatment with insulin, pregnancy, drug abuse, alcohol abuse, untreated sleep apnoea, previous cardiovascular event, diabetes complications (proliferative retinopathy, renal failure stadium 3, symptomatic neuropathy) as well as any other condition which in the opinion of the investigator would render the patient unsuitable for inclusion in the study. We assessed anthropometric data and asked the patients to fill out HRQoL questionnaires (RAND SF-36), at baseline, 6 months, and 2 years. At 2 years, interviews were performed for an in-depth analysis of HRQoL changes. The patients for this prospective study were recruited from the Department of Endocrinology at the University Hospital in Uppsala, Sweden, and were patients with T2D that were scheduled to undergo RYGB according to national guidelines between 2015–2017 and followed up until 2019.

### RAND SF-36

The RAND SF-36 health questionnaire assesses eight separate health concepts with multi-dimensional scales. These are physical functioning, role limitations due to physical health problems, pain, general health, energy/fatigue, social functioning, role limitations due to emotional health problems, and emotional well-being. An additional single item assesses change in perceived health during the last 12 months. Scoring the questionnaire is a two-step procedure; first, for each item, the response category is recoded to a numeric value according to a scoring key, and second, all specific items in the same subscale are averaged to create the 8 subscale scores. The scores may be treated as an ordinal scale or as an approximation of an interval or ratio level scale. Scores in subscales range from 0 to 100, with 100 representing the best level of health status [18]. A difference of 3–5 points in the RAND-36 subscales is considered clinically important [19, 20]. However, no overall total score is calculated. The scale in this study was treated as an interval level scale.

### Qualitative interviews

The interviews were conducted 2 years after the RYGB surgery by a social worker. All participants were invited to the interview in April 2019. Eleven of them were interviewed at the hospital´s outpatient department in a quiet room, while two were held by telephone.

At the beginning of each interview, a general introduction of the study and an overview of the purpose of the study/ or with the interviews and its confidentiality were provided. The duration of each interview varied between 0.5 and 1.5 h, and all participants were given enough opportunity to share their views. A semi-structured interview guide was developed according to the aim of the study. Furthermore, probing questions were used, e.g., “Could you please further describe the situation using a concrete example?” All interviews were audio-recorded with a digital voice recorder and transcribed verbatim by a medical secretary.

Table 1 shows the interview guide.

**Table 1:** Interview guide used during the 2-year follow-up after metabolic surgery

Can you tell me how you feel today?
Can you tell me about how you lived your life today?
Can you tell me about how you view the expectations you had before the surgery and how you feel about them now?
How has your physical health been affected by the operation? How has your psychological life been affected by the surgery?
How has your social situation been affected by the operation? Has your life been changed by the surgery? If so, in what way?
Has something gotten better in your life? Has anything gotten worse in your life?
What challenges have you faced after the surgery and how have you handled them?
Has your view of yourself been affected by the operation? If so, in what way?
Has other people's views of you been affected by the operation? If so, in what way?
How do you feel about the time before surgery now? How do you feel about the surgery now?
How do you feel about the support you received after the surgery? When do you think of your future when it comes to your living habits?
Is there anything you want to add?

#### Qualitative content analysis

A qualitative content analysis, inspired by Krippendorff [21], was used with an inductive approach. The interviews and content analysis were performed in Swedish. A professional translator has translated the quotations used in this paper into English.

Data analysis was performed according to the following four steps:

1. The transcribed individual interviews were read through several times to obtain a sense of the whole. After discussion, it was agreed that saturation had been reached.
2. The transcribed text was divided into units of meaning, which were condensed and labeled with codes and discussed in the research group (PK, ER, and JL). Also, two of the authors (ER and JL) reflected, discussed, and verified that the coding was congruent with the units of meaning.
3. The various codes were compared, and similarities and differences were identified, and then sorted the codes into subthemes. These subthemes are revealed in three main themes. (By consensus among all authors).
4. The theme was carefully assessed based on internal homogeneity (ie, data belonging to the same theme were judged to be related in a meaningful way) and external heterogeneity (i.e., the categories were distinguishable in that the differences among them were clear).

The analysis was based on a manifest interpretation of the text, three of the authors made a latent interpretation of the content analysis, and the overarching latent theme was revealed.

#### Statistical analysis

Categorical data are presented as frequencies and percentages, *n* (%), while continuous data are given as means ± standard deviations (SDs). Wilcoxon signed rank tests were used for examining changes in the RAND-36 domains from baseline to 6 months and 2 years follow-up. Multiple linear regression models were used to assess possible associations between decrease in weight, BMI, and HbA1c from baseline to 6 months and 2 years follow-up and change in the RAND-36 domains during the same periods. Statistical analyses were performed in R 3.6.2/4.0.0 (R Foundation for Statistical Computing, Vienna, Austria), with p-values < 0.05 considered statistically significant.

## Results

We recruited 14 patients (55 ± 9 years, 10 women) with a mean T2D duration of 4 ± 3 years. One patient was excluded due to an eating disorder. At baseline, HbA1c was 55 ± 12 mmol/mol and patients had a body mass index (BMI) of 36.8 ± 4 kg/m^2^, corresponding to a weight of 99.8 ± 13.7 kg. The expected beneficial metabolic effects, including large weight loss and improved glycaemic control, were obtained and have already been reported [16, 17]. Briefly, BMI decreased to 28.5 ± 3.2 at 6 months and was maintained at that level (28.6 ± 3.5) 2 years after surgery. HbA1c levels decreased to 41 ± 6 at 6 months and remained stable at 2 years (41 ± 5).

### Health-related quantitative assessment (RAND-36)

**Fig. 1:**
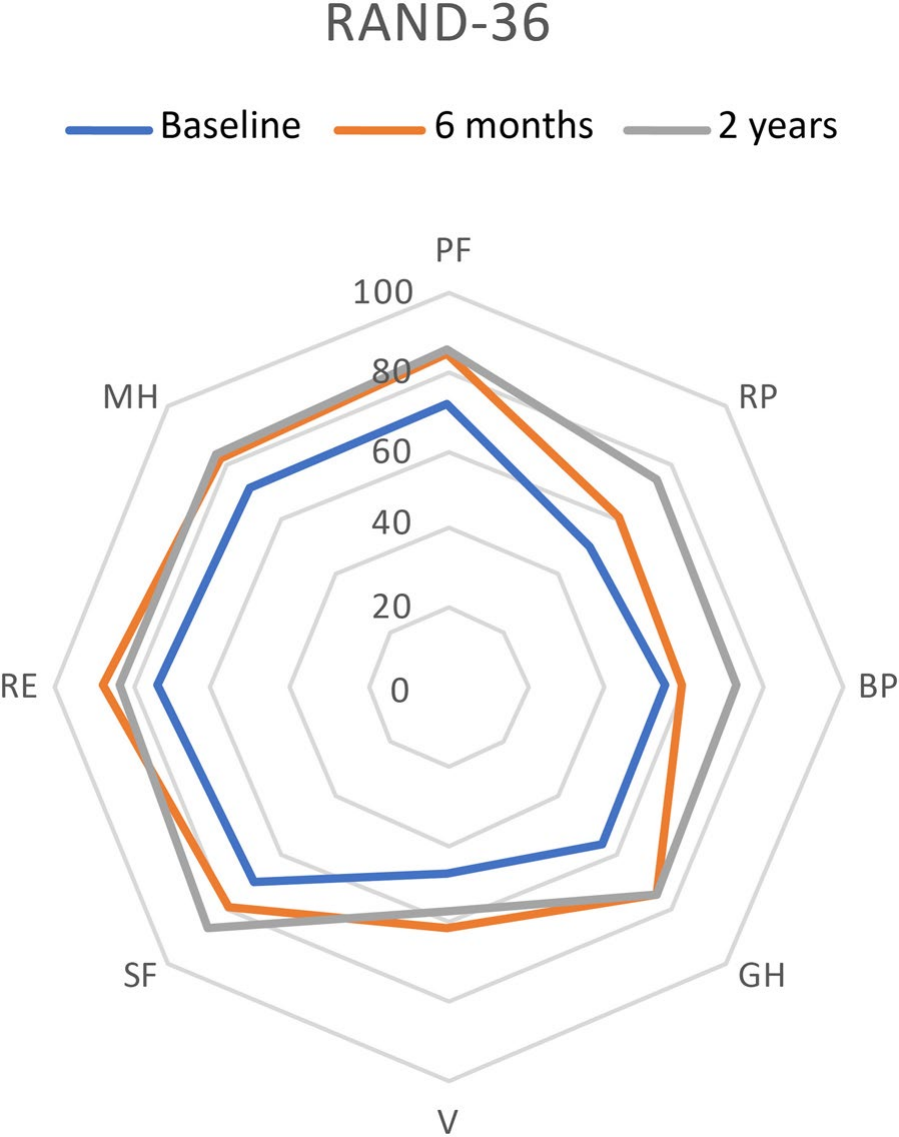
Results for RAND-36 domains at baseline and follow-up at 24 weeks and 2 years

**Fig. 2:**
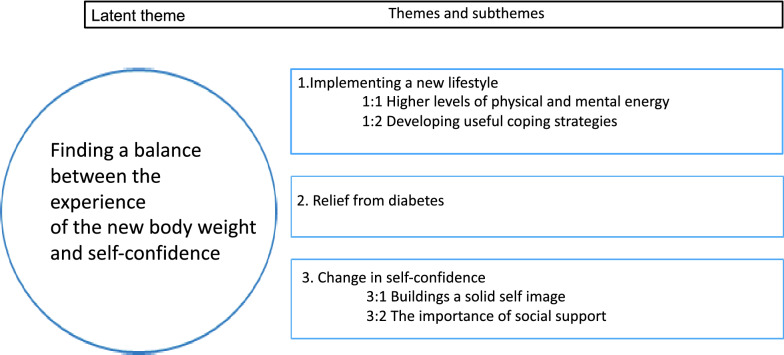
Latent theme themes, subthemes

At 6 months, a significant improvement in HRQoL was seen in general health (*p* = 0.019) and emotional well-being (*p* = 0.040). At 2 years, improvements were seen in physical functioning (*p* = 0.028), general health (*p* = 0.013), energy/fatigue (*p* = 0.032), and emotional well-being (*p* = 0.008) (Figs. 1 and 2).

### Multiple regression analysis

Exploratory regression analyses were performed to elucidate whether the magnitude of changes in patient characteristics from baseline to follow-up predicted improvements in HRQoL measures. Surprisingly, the degree of weight loss was inversely associated with the improvement in the RAND-36 domains BP and EF at 6 months. Otherwise, there were no significant associations for weight, BMI, and HbA1c changes from baseline to follow-up (Table 2). Thus, weight loss per se could not explain the improved HRQL measures.

**Table 2:** Results from multiple linear regression models of the effect on change in SF-36 domains from baseline to 6 months and 2 years (outcomes) of a one-percentage-point decrease in weight, BMI, and HbA1c from baseline to 6 months and 2 years (predictors)

Outcome	Predictor	6 month follow-up		2 year follow-up	
		Adjusted β (95% CI)^b^	*P* value	Adjusted β (95% CI)^b^	*P* value
ΔPF	Weight decrease (%)	− 2.24 (− 5.63; 1.16)	0.171	− 0.01 (− 1.55; 1.52)	0.983
ΔRP	Weight decrease (%)	− 4.60 (4.01; − 1.21)	0.257	0.12 (− 4.40; 4.64)	0.952
ΔBP	Weight decrease (%)	− 4.43 (− 8.54; − 0.32)	*0.037*	0.40 (− 3.71; 4.51)	0.829
ΔGH	Weight decrease (%)	− 3.55 (− 8.38; 1.30)	0.131	− 1.60 (− 4.50; 1.30)	0.239
ΔVT	Weight decrease (%)	− 2.50 (− 6.90; 1.94)	0.236	− 1.36 (− 3.10; 0.39)	0.111
ΔSF	Weight decrease (%)	0.71 (− 2.49; 3.91)	0.626	− 1.99 (− 4.51; 0.52)	0.105
ΔEF	Weight decrease (%)	− 5.15 (− 8.25; − 2.04)	*0.005*	− 0.40 (− 3.97; 3.16)	0.801
ΔMH	Weight decrease (%)	− 0.68 (− 3.20; 1.83)	0.553	− 0.13 (− 1.10; 0.84)	0.770
ΔPF	BMI decrease (%)	− 2.12 (− 5.12; 0.88)	0.144	0.14 (− 1.27; 1.55)	0.824
ΔRP	BMI decrease (%)	− 6.83 (− 15.00; 1.33)	0.091	0.60 (− 3.62; 4.81)	0.753
ΔBP	BMI decrease (%)	− 6.34 (− 10.92; − 1.76)	*0.012*	0.45 (− 3.41; 4.31)	0.795
ΔGH	BMI decrease (%)	− 2.53 (− 6.97; 1.92)	0.230	− 0.53 (− 4.05; 1.46)	0.309
ΔVT	BMI decrease (%)	− 3.42 (− 7.50; 0.65)	0.900	− 1.20 (0.56; − 1.57)	0.154
ΔSF	BMI decrease (%)	0.11 (− 3.25; 3.48)	0.941	− 2.14 (− 4.47; 0.20)	0.067
ΔEF	BMI decrease (%)	− 3.31 (− 6.75; 0.14)	0.058	− 0.17 (− 3.47; 3.13)	0.908
ΔMH	BMI decrease (%)	− 0.73 (− 2.90; 1.44)	0.467	− 0.14 (− 1.04; 0.77)	0.740
ΔPF	HbA1c decrease (%)	− 1.95 (1.81; 1.42)	0.791	− 0.17 (− 1.04; 0.70)	0.666
ΔRP	HbA1c decrease (%)	-1.80 (1.16; 1.8)	0.34	−2.99 (-1.70; 2.62)	0.025
ΔBP	HbA1c decrease (%)	− 0.78 (− 3.42; 1.85)	0.518	0.09 (− 2.83; 3.01)	0.073
ΔGH	HbA1c decrease (%)	− 0.69 (− 2.98; 1.60)	0.512	0.01 (− 2.22; 2.20)	0.996
ΔVT	HbA1c decrease (%)	− 1.05 (− 2.56; 0.45)	0.145	− 1.05 (− 2.56; 0.45)	0.145
ΔSF	HbA1c decrease (%)	− 0.94 (− 2.43; 0.55)	0.190	− 1.91 (− 3.91; 0.08)	0.058
ΔEF	HbA1c decrease (%)	4.98 (− 1.25; 2.24)	0.988	1.61 (− 0.98; 4.20)	0.189
ΔMH	HbA1c decrease (%)	− 0.57 (− 1.45; 0.31)	0.177	0.245 (− 0.48; 0.97)	0.460

Italic indicates significant *p* values

^a^Change from baseline to follow-up in Physical functioning (PF), Role functioning/physical (RP), Pain (BP), General health (GH), Energy/fatigue (VT), Social functioning (27), Role functioning/emotional (EF, and Emotional well-being (MH)

^b^Adjusted for values of outcome and predictor variables at baseline

#### Qualitative evaluation

##### Finding a balance between the experience of the new bodyweight and self-confidence

The latent theme “Finding a balance between the experience of the new bodyweight and self-confidence” was built upon the three themes “The implementation of a new lifestyle”, “The relief from diabetes” and “The change in self-confidence” (Fig. 2).

##### Implementing a new lifestyle

*Higher levels of physical and mental energy* After surgery, participants expressed that it was a process to implement a new lifestyle, which included doing physical activities, developing new eating habits, and establishing better control of their eating behaviors. The participants highlighted higher levels of physical and mental energy related to the ease of being physically active after weight loss.

“I said it, it was probably not just the stomach they operated on; they probably took some bite in my head as well. You’ve found things that you’ve come back to. Sewing, I did before. I crocheted a lot. Now I crochet again. When you lose weight, you get a little more energy. For eating regularly and healthily, you get that energy too” (Patient 10).

“My obesity made me barely able to walk when I was heaviest. Now I walk easily and I will not take the elevator anymore. And I walk easily for several hours” (Patient 2).

*Developing useful coping strategies* The new lifestyle enabled an active professional life with a reduction in sick leave. Several participants returned to working life with extended working hours.

“The number of sick days has decreased. I have become much healthier because I was very sick before, too. So, I met my boss who went through the statistics and says that there is a big difference” (Patient 8).

Nevertheless, participants described both external and internal inhibitory factors for the new lifestyle they faced, and this required an ability to cope. They described external factors as situations that sometimes caused trouble and difficulties with the size of food portions. This generated problems, especially during travel or on special occasions such as Christmas. Christmas time or other social occasions at work caused problems. There were difficulties in ordering little food at restaurants:

“I want a children's portion, or I want a tiny bit of something. Often there is no children's menu. That’s a pity” (Patient 1).

“We were on a cruise last autumn, a Mediterranean cruise. And I, as I said, I ate everything they served but less of it. But the staff were so desperate because they didn't think I liked the food. Because I always left food. And then they said, we'll get something else. Don't you want it? We will get something new. No, no” (Patient 12).

Internal factors for several participants were stomach pain and, for some, difficulties with dumping and having moments of fatigue. These symptoms were alleviated partly by the participants learning to manage the symptoms and partly over time.

“Yes, I think it took a very long time to learn new habits before getting it right. For the first half of the year, there were probably quite a few times I felt bad or had a little stomach crunch. Now it's probably nothing … No, it's been a long time now” (Patient 4).

“I have iron deficiency, zinc deficiency, and vitamin D-deficiency. So, I felt bad because of that. So, I've been… well, at first, I got very energetic after the surgery, very fast. But then the iron deficiency came pretty quickly, so then I got very tired … Even though I have had problems with iron and zinc, and I have been tired, I still think the operation is worth it in some way” (Patient 3).

##### Relief from diabetes

The participants expressed many thoughts and feelings related to diabetes. In particular, they talked about doing something good and valuable to the body. Besides, participants expressed a feeling of freedom. Freedom from taking medications as well as freedom from illness in addition to diabetes, such as heart failure and high blood pressure. The freedom itself led to a better general quality of life.

Yes, all my illnesses. I had diabetes, high blood pressure, blood lipids, medication for everything. And I don't have anything like that now. I did a check, like a conclusion. Also with this, they took blood and tested everything then. And nothing came back, so to speak. Completely gone. (Patient 12).

I guess that’s, I mean not having diabetes, what I was hoping for, as well as with the surgery… I just think getting rid of my type 2 diabetes has been worth it all. I have a much better quality of life (Patient 6).

##### Change in self-confidence

*Building a solid self-image* Participants described changes in how they saw themselves (self-confidence/image) after surgery and expressed a positive belief in the future. Descriptions of self-confidence were expressed using a “then and now” comparison, and describing how well their present body image matched their real bodies, i.e., how the participants actually looked. Furthermore, support from significant others was of vital importance in building self-confidence.

“I have had a picture in the head of a rather large hippopotamus. When you look in the mirror, I’ve said, ‘Who is that?’ … When I started to get my chin back, I could do this all the time because this has not been visible. It was very thick under here. And God, I have a chin! … So my self-image does not match how I look. I am drawn with the old self-image but have started to catch up” (Patient 2).

“Yes, I feel that sometimes when I sit and think that there was so much fat around this part of the brain … maybe there was also a fat lump there. Now I see the future more clearly” (Patient 1).

”I feel great, that’s the best thing I’ve done. I wish I could have done this ten years ago. So, I am fine, no complications…I had type 2 diabetes of course, and now I have normal values” (Patient 11).

"You had a sense of shame many times. I tried to hide, so to speak, in certain clothes and things like that. And that feeling has not completely disappeared but is at least much, much better in that way, than that I dressed in a certain way. You never put down a shirt for example and it's kind of no problem in today's situation…Well, I've had one gone through a journey so to speak, to get there where I am now. I feel good when I look in the mirror today” (Patient 12).

*The importance of social support* The importance of social functioning, via support from friends, family, working places, and the health care personnel, was highlighted. The participants did not have to explain their situation to those who already knew. They also mentioned that they participated more in social activities, which affected their quality of life.

“The support from those around me has been positive. No one has said anything negative. They have been very understanding. Workmates at work have been perfect" (Patient 1).

“It's just been positive with the surroundings, it's actually been. No one has said like, no but now you cannot lose weight anymore, or you look sick or… They've been really appreciative. The job has been perfect. They told me, but you, I think it's time for you to eat. Well, that's right. My friends, too, there has been a very great understanding. No one who has thought I've done anything strange" (Patient 10).

Another highlighted that their social life had improved: “I have better health, I think. And not just health, but also my body and social life and so on. Yes, socially then, the quality of life has become much better” (Patient 9).

## Discussion

The results showed a significant improvement in general health and emotional wellbeing already at 6 months after surgery. In addition, after 2 years, improvement in four of the eight HRQoL domains was found (physical functioning, general health, energy/fatigue, and emotional well-being). The analysis from the qualitative interviews had the latent theme of “Finding a balance between the experience of the new bodyweight and self-confidence”, which was built upon three themes: “implementation of a new lifestyle”, “relief from diabetes” and “change in self-confidence”.

Previous studies have shown that before surgery, obese patients estimate their mental health to be lower than normal-weight people [7]. This may contribute to an increased risk for psychosocial problems post-operatively. In our study, the participants scored lower mental health in comparison with what was found in a study among patients withT2D in Sweden [22].

Earlier studies demonstrated mixed results concerning HRQoL postoperatively [7, 13]. In the present study, the patients scored higher in six of the eight domains of the RAND-36 instruments when compared at 2 years. All improvements were well above 3–5 points, i.e., of significant clinical importance. Moreover, in comparison with a randomized sample of 1353 Swedish patients with T2D, the present scores were higher in physical functioning (86.3 vs 70.6), less pain (72.8 vs 62.5), general health 75.4 vs 60.8), social functioning (83.0 vs 74.6) and mental health (83.0 vs 74.6). Age, lower preoperative BMI, male gender, higher education, professional status, and disposable income were associated with a higher postoperative HRQoL. [23].

Ten years after gastric bypass, operated patients showed better scores in most aspects of HRQoL compared to obese controls but did not achieve the levels of the general population [24].

Interestingly, the analysis of the qualitative interviews confirmed the results from the RAND-36. This confirmation could be explained in the latent theme of “Finding a balance between the experience of the new bodyweight and self-confidence”, which indicates that it was not necessarily the weight loss that contributed to the improvement in the HRQoL. Instead, the participants expressed that it was a learning process to establish new eating habits, become more bodily active, and take part in social life. In that, finding a balance between the reduced body weight and increased self-confidence was crucial. In addition, the participants expressed that they had done something good for their body by relieving themselves of diabetes. Similar results have been described by Ogden et al. [7] as well as by Liebl et al. [25]. A future success factor could be to involve the patients in their responses to the PROM form (in this study HRQoL), making it more visible how life has changed after bariatric surgery. One proposal is to develop digital PROM forms, in which the patients continuously can take part in their own improvement in quality of life [26].

Several limitations must be acknowledged. Firstly, the study was not powered to detect associations between anthropometric data and changes in HRQoL. However, despite the small number of included patients (n = 13), significant improvements could be demonstrated in 6 of the 8 subscales at 2 years. Secondly, no disease-specific questionnaires, such as the Obesity Problems Scale (OP), were used to evaluate the influence of specific weight-related factors. Nevertheless, the present large improvement in general quality of life implies a strong association between metabolic surgery and improved HRQoL in obese patients with T2D. Thirdly, although the answers from the interviews were very homogeneous, the fact that some people possess the power of the word and express themselves in a clear way could lead to an unbalanced presentation of comments.

Regarding external validity, the small number of studied individuals makes it difficult to generalize. However, as our patients demonstrated the typical characteristics of individuals with T2D having bariatric surgery in Sweden (female dominance, diabetes since a couple of years and a rather moderate BMI), we believe that the result can be transferred to similar patient groups. Here we want to reconnect once again with how qualitative and quantifiable data can generate a more holistic picture of the result. The importance of following this patient group over time provides new knowledge about how life portrayed after bariatric surgery. In particular, we would like to stress the value of the fact that there are factors other than weight loss that can affect patients' HRQoL in the post-operative process. The study demonstrates the value of the fact that by the participants putting into words, it leads to a richer description than simply evaluating numbers. Based on the results of the qualitative study, it is not only the weight loss itself that is important for a good HRQoL, it is also required that the patient finds a balance between weight loss and self-confidence. It would be of value as a focus of a future larger study, to develop and evaluate a specific PROM regarding the named balance in the bariatric context.

## Conclusion

Our study provides insight into the psychosocial experience of patients with type 2 diabetes after RYGB. The interview part of the study was invaluable because it provided insight into how the patients experienced the whole procedure from the pre-operative period to the post-operative period. Asking patients to describe their experience provides valuable information to health providers for more personalized discussions and customized guidance throughout the process.

## Data Availability

The datasets used and/or analyzed during the current study are available from the corresponding author on reasonable request.
